# Dehydroepiandrosterone, Cancer, and Aging

**DOI:** 10.14336/AD.2021.0913

**Published:** 2022-04-01

**Authors:** Arthur G Schwartz

**Affiliations:** Fels Institute for Cancer Research and Molecular Biology and Department of Microbiology, Lewis Katz School of Medicine at Temple University, Philadelphia, PA 19140, USA.

**Keywords:** DHEA, G6PD, NADPH, NOX, Cortisol

## Abstract

The biological significance of dehydroepiandrosterone (DHEA) which, in the form of its sulfated ester is the most abundant steroid hormone in human plasma, is an enigma. Over the past years, numerous investigators have reported preclinical findings that DHEA has preventive and therapeutic efficacy in treating major age-associated diseases, including cancer, atherosclerosis, diabetes, obesity, as well as ameliorating the deleterious effects of excess cortisol exposure. Epidemiological studies have also found that low DHEA(S) levels predict an increased all-cause mortality. However, clinical trials, in which oral doses of DHEA at 50 mg-100 mg have been administered to elderly individuals for up to two years, have produced no clear evidence of benefit in parameters such as body composition, peak volume of oxygen consumption, muscle strength, or insulin sensitivity. I discuss why clinical trials, which use doses of DHEA in the 100 mg range, which are the human equivalent of about 1/20^th^ the doses used in animal studies, are an inadequate test of DHEA’s therapeutic potential. I also discuss three mechanisms of DHEA action that very likely contribute to its biological effects in animal studies. Lastly, I describe the development of a DHEA analog which lacks DHEA’s androgenic and estrogenic action and that demonstrates enhanced potency and is currently in clinical trials. The use of such analogs may provide a better understanding of DHEA’s potential therapeutic utility.

Of the six primary steroid hormones in humans, estradiol, progesterone, testosterone, cortisol, aldosterone, and DHEA(S), the biological role of DHEA(S) remains an enigma. In humans the plasma levels of DHEA(S) peak in the second decade of life and thereafter decline continuously to 5%-10% of their maximum in the 8^th^ to 9^th^ decade [1, 2]. Epidemiological studies have found that low serum levels of DHEA(S) are associated with lower physical vitality and function [3] as well as an increased cardiovascular and all-cause mortality [2, 4, 5].

Over the past decades, numerous preclinical studies by different investigators have reported that DHEA has preventive and therapeutic efficacy against major age-associated diseases. These include cancer [6-13], atherosclerosis [14-16], diabetes [17, 18], obesity [19, 20], as well as amelioration of the deleterious effects of excess glucocorticoid exposure [21]. However, clinical trials, in which DHEA was administered orally daily at doses of 50mg-100 mg, which restores the plasma levels of DHEA(S) in the elderly to peak levels in the second decade, produced no apparent beneficial effect [22].

How do we account for the discrepancy between the preclinical and epidemiological findings and the results of the clinical trials? In the following, I discuss three biochemical-physiologic actions of DHEA that very likely contribute to its preventive and therapeutic efficacy in preclinical studies as well as a possible explanation for the lack of clinical efficacy when 50 mg-100mg is administered to elderly individuals. I also describe the development of a non-androgenic analog of DHEA with enhanced potency and suggest that the testing of such analogs through non-oral administration might advance our understanding of the possible benefits of such compounds in ameliorating the development of age-related disease.

## DHEA, Oxidative Stress, NADPH Oxidase, and Diseases of Aging

There is increasing evidence that low-level, chronic inflammation and its associated oxidative stress plays a central pathophysiologic role in the development of age-related diseases [23,2 4]. There are various enzymatic sources of reactive oxygen species (ROS), including xanthine oxidase, cytochrome p450, nitric oxide synthase, and the NADPH oxidases (NOX), a group of seven widely distributed enzymes, whose only known function is the production of ROS [25]. The first of these enzymes to be discovered, NOX 2, is the classical phagocyte NOX responsible for the respiratory burst and noted for its microbiocidal activity.

**Figure 1.**
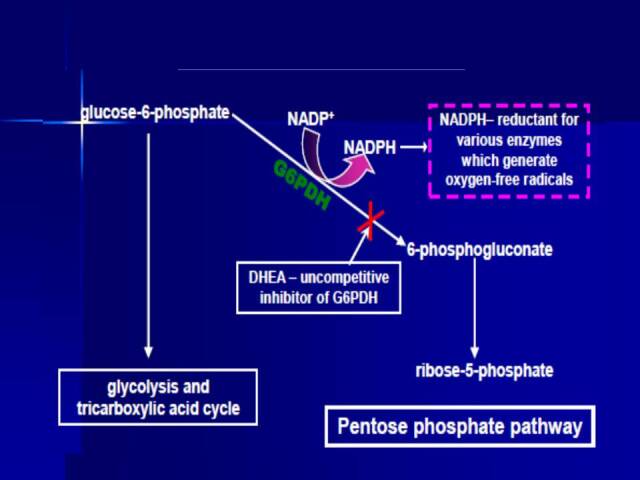
The inhibition of G6PD and NADPH production by DHEA and related steroids. DHEA is a potent uncompetitive inhibitor (with respect to NADP+ and glucose-6-phosphate) of mammalian G6PD and thereby reduces the availability of NADPH and generation of reactive oxygen species by NADPH-dependent enzymes. Reprinted from Schwartz, AG, Pashko, LL, “Dehydroepiandrosterone, glucose-6-phosphate dehydrogenase, and longevity,” Aging Research Reviews, 2004, 3, 171-187, by permission of Elsevier.

Numerous studies suggest that the NOX enzymes appear to play a central pathophysiological role in the development major age-associated diseases, including cancer [26], atherosclerosis [27], ischemic stroke [28] as well as fibrotic [29] and neurodegenerative disease [30]. As discussed below, DHEA inhibits NOX activity through the inhibition of glucose-6-phosphate-dehydro-genase (G6PD), the major source of cytosolic NADPH [31], which is an obligatory substrate for NOX activity [32]. Inhibition of G6PD is primarily responsible for the inhibition of skin tumor promotion by DHEA and structural analogs and very likely contributes to its cancer preventive and anti-atherosclerotic action in other experimental models (Fig. 1).

## Glucose-6-phosphate Dehydrogenase Inhibition and Two-stage Skin Tumorigenesis

DHEA is a potent uncompetitive inhibitor of mammalian G6PD [33] and as a consequence inhibits NOX-dependent ROS production in various cell types. These include tetradecanoylphorbol-13-acetate (TPA)-stimulated neutrophils [34], lipopolysaccharide-stimulated microglial cells [35], and high glucose-stimulated adipocytes [36]. As described below, this mechanism very likely accounts for the inhibition of TPA-promoted skin tumor formation in mice. In addition, DHEA also inhibits the activity of the NADPH-dependent cytochrome p450 which converts chemically inactive carcinogens such as 7,12-dimethylbenz (a)anthracene (DMBA) to their carcinogenic forms [37, 38]. As a consequence, DHEA also inhibits the tumor initiation process as well.

The two-stage skin tumorigenesis model is an extensively studied in vivo model of the sequential development of epithelial tumors [39]. In this model, tumor initiation occurs following the topical application of a sub-carcinogenic dose of carcinogens such as DMBA. DMBA undergoes conversion by the NADPH-cytochrome p450 to a reactive DNA-binding form, which produces activating mutations in the *Hras 1* gene [40]. Repeated application of a tumor promoter produces a sustained epidermal hyperplasia leading to selective growth of initiated cells into papillomas [39].

No tumors appear until the DMBA-treated skin is treated repeatedly with a tumor promoter, such as TPA. TPA is perhaps the most widely used diacylglycerol mimetic and activator of protein kinase C-mediated actions, including NOX activation via phosphorylation of p47-*phox* [41]. The resultant NOX-generated ROS leads to a sustained inflammatory and epidermal hyperplastic response and the appearance of multiple squamous papillomas within a few months. Either oral administration or topical treatment with DHEA or non-androgenic structural analogs singly before DMBA application, or repeatedly before each TPA application, inhibits papilloma development [9, 10]. DHEA and analog treatment inhibited the rate of binding of (3)H-DMBA to mouse skin DNA, very probably by reducing NADPH-dependent cytochrome p450 activation of the DMBA, and this likely accounts for the anti-initiating action of these steroids [9,42].

A single application of the DHEA analog 16α-fluoro-5-androsten-17-one (fluasterone), a more potent G6PD inhibitor (Ki of fluasterone is 0.51 uM vs. 18.7 uM for DHEA), to mouse skin before TPA treatment suppressed the TPA-induced acute inflammatory and epidermal hyperplastic effect [43]. The endogenous glucocorticoid in mice, corticosterone, which does not inhibit G6PD but exerts anti-inflammatory action through interaction with the glucocorticoid receptor, was as effective as fluasterone in suppressing inflammation and hyperplasia. Importantly, intradermal injection of a mixture of NADPH and cationic liposomes (to enable cellular uptake of the normally impenetrable dinucleotide) reversed the anti-inflammatory and anti-hyperplastic effect of fluasterone but had no apparent effect on corticosterone action [43].

We also found that treatment of mice with a mixture of the four deoxyribonucleosides of adenine, guanine, cyotosine and thymine (DRN) in the drinking water reversed the anti-inflammatory and anti-hyperplastic effect of fluasterone in acute experiments as well as completely reversed the inhibition of TPA promotion of papilloma development by fluasterone [44]. DRN provision might restore nucleotide pools that are reduced through inhibition of ribose-5-phosphate production, the synthesis of which might also be reduced by G6PD inhibition, as well as increase the availability of NADPH, which is no longer required for the synthesis of purine and pyrimidine nucleotides [31]. A similar protective effect of DRN administration was found by Garcea *et al*. in a model of diethylnitrosamine-initiated and phenobarbital-promoted liver tumorigenesis in rats. These investigators found that dietary DHEA administration during the promotion phase reduced the development of liver preneoplastic foci, and intraperitoneal injection of DRN for 12 days reversed the protective effect of DHEA [45].

The findings that both NADPH-liposomes as well as DRN treatment reverses the fluasterone inhibition of TPA-induced inflammation, epidermal hyperplasia, and papilloma development strongly suggest that inhibition of G6PD by fluasterone is critical to its anti-tumor promoting action.

## Anti-glucocorticoid Action of DHEA

Prolonged excess cortisol exposure, as occurs in patients with endogenous or exogenous Cushing’s syndrome, produces numerous untoward effects, including weight gain, hyperglycemia, hypertension, immunosuppression, myopathy, osteoporosis, and thinning of the skin [46]. DHEA has been shown to antagonize many of these untoward effects of glucocorticoid (GC) exposure in mice and rats [21]. Many of the morbidities associated with excess glucocorticoid occur naturally with age, and since cortisol levels increase with age [47,48], whereas DHEA(S) levels decline precipitously, this has led to the hypothesis that the change in these hormone levels is causally linked to GC-associated-morbidity development [49].

**Figure 2.**
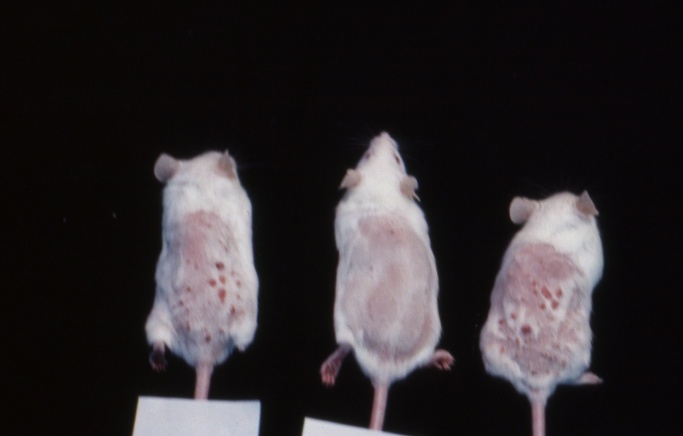
Reversal of fluasterone inhibition of TPA promotion of skin papillomas by deoxyribonucleoside (DRN) administration. Color photograph of one mouse from the TPA (left), fluasterone + TPA (center), fluasterone + TPA + DRN (right) treatment groups. The picture was taken after 64 days of TPA treatment, and each animal represented the mouse with the greatest number of tumors in its respective group at that time: TPA, 24 tumors; fluasterone + TPA, 4 tumors; and fluasterone + TPA + DRN, 26 tumors. Mice were anesthetized with Metofane (not euthanized) prior to taking pictures. Reprinted from “Pashko, LL, Lewbart, ML, Schwartz, AG, “Inhibition of 12-O-tetradecanoylphorbol-13-acetate-promoted skin tumor formation in mice by 16a-fluoro-5-androsten-17-one and its reversal by deoxyribonucleosides,” Carcinogenesis, 1991, 12, 2189-2192, by permission of Oxford University Press.

One of the adverse effects of GC’s is immune-suppression, and this is demonstrated acutely by the reduction in thymus and spleen weights following high dose GC treatment. Pretreatment of mice for three days with DHEA (60 mg/kg sc) antagonized the suppression of *in vitro* blastogenic responses in T- and B-lymphocytes observed after a single injection of dexamethasone (Dex; 60 mg/kg sc) as well as protected against thymic and splenic atrophy [50]. This protection against Dex-induced thymic and splenic atrophy has clinical significance: Ben-Nathan and co-workers reported that Dex treatment of mice, in addition to causing thymic and splenic atrophy, greatly increased susceptibility of mice to mortality following ip injection with an attenuated strain of West Nile Virus from 0% to 67% over a 21-day period. Treatment with DHEA both protected mice against thymic and splenic atrophy and significantly reduced mortality from 67% to 11% [51]. In other models of glucocorticoid-induced adverse effects in rats, DHEA protected against Dex-induced hypertension as well as prednisolone-induced osteoporosis [52, 53] (Fig. 2).

It is likely that the anti-obesity and anti-diabetic effects of DHEA are also mediated through its anti-GC action. The non-androgenic DHEA analog, fluasterone, in addition to being a more potent G6PD inhibitor, is also about 6X as potent in protecting mice against Dex-induced thymic and splenic atrophy [54]. When eight synthetic and natural DHEA analogs, related to fluasterone but containing different substituents in the 3β and 16α positions, were screened for activity in protecting against Dex-induced thymic and splenic involution, fluasterone was the most potent steroid tested [54]. Interestingly, there was a one-to-one correlation between potency in protecting against thymic and splenic involution and potency in reducing weight gain, indicating that the anti-GC activity of these steroids is mechanistically linked to their anti-obesity action (Schwartz, A, Pashko, L, unpublished observation).

The mechanism of the anti-GC effect of DHEA is not known. DHEA does not bind to the GC receptor and is not a competitive inhibitor [55]. DHEA, both in vitro in cultured adipocytes and *in vivo* in mouse adipose tissue and liver, down regulates the expression and oxoreductase activity of 11β-hydroxysteroid dehydrogenase type 1 (11β-HSD1), the enzyme which locally reactivates GC’s [56]. 11β-HSD1 knockout mice are resistant to GC-induced side effects, such as hyperglycemia, myopathy, thinning of skin, *etc*. [57]. However, inhibition of 11β-HSD1 by itself cannot account for DHEA’s protection against Dex-induced thymic and splenic atrophy, since Dex does not require activation by 11β-HSD1, and 11-keto Dex is as potent a glucocorticoid receptor agonist as is Dex [58]. The receptor mediating the anti-GC action of DHEA, as well as its mechanism of action, has not been identified.

Treatment of mice or rats with DHEA produces hepatomegaly and peroxisome proliferation, presumably through activation of peroxisome proliferator activated receptor alpha (PPARα) [59,60]. Also, DHEA treatment of mice up-regulated hepatic expression of peroxisome proliferator activated receptor gamma (PPARγ), the critical receptor for the anti-diabetic thiazolidinediones [56]. It has been suggested that the peroxisome-proliferator action of DHEA may contribute to some of its metabolic and cancer preventive effects [60]. However, fluasterone, when administered to mice at doses which produce anti-obesity, anti-diabetic, and cancer preventive effects, did not induce hepatomegaly or stimulate liver catalase activity [42]. On the contrary, both DHEA and clofibrate, a known peroxisome proliferator, at comparable doses significantly enhanced liver weight and catalase activity [42]. Further work is required to determine the significance of PPARα and PPARγ activation in the therapeutic effects of the DHEA steroids.

## Estrogen Receptor Beta Agonism

Estrogen receptor alpha (ERα) and estrogen receptor beta (ERβ) both belong to the family of nuclear steroid receptors which share a common four-unit structure and act as ligand-activated transcription factors. ERα was believed to be the sole ER until a second receptor (ERβ) was cloned from rat prostate in 1996 [61]. The ligand specificities of ERα and ERβ are sufficiently different that agonists specific to each receptor have been developed [62]. Employing such agonists, it has been demonstrated that ERβ activation does not lead to some of the classical estrogen hormonal actions, such as uterine and breast epithelial hyperplasia, which are mediated by ERα. ERβ activation, on the contrary, has been shown to antagonize the proliferative effects of ERα activation in the mammary glands of rats [63].

Importantly, there is considerable evidence that Erβ acts as a broad-spectrum ligand-activated tumor suppressor. In cancers of the colon, ovary, and prostate, loss of ERβ expression is associated with higher cancer stages and a reduced overall survival [64-66]. Bossard et al., utilizing an ovarian cancer cell line derived from a patient with a stage III poorly differentiated adenocarcinoma, which expressed high endogenous levels of ERα but little ERβ, found that adenovirus reintroduction of ERβ led to an inhibition of both basal and estradiol-induced cell proliferation in vitro. Importantly, ERβ reintroduction reduced the growth and metastatic spread of these cells when injected into mice in an orthotopic xenograft model and led to increased survival [67]. Other investigators, using human cell lines derived from prostate cancer [68], gliomas [69] as well as T- and B-cell lymphomas [70], all of which expressed detectable levels of ERβ, found that treatment with ERβ specific agonists inhibited cell proliferation *in vitro* as well as growth and metastatic spread in xenograft in vivo models.

Chen et al. reported that DHEA binds to and transcriptionally activates ERβ with “an approximate 5-10-fold higher transcriptional specificity for the ERβ receptor *vs*. ERα” and “has the potential for physiologically relevant direct activation of ERβ” [55]. Saijo et al. found that ERβ-specific ligands mediate the recruitment of CtBP corepressor complexes to AP-1 and thereby repress inflammatory gene expression via a transrepression mechanism in microglial cells [71]. They identified 5-androsten-3β,17β-diol, a DHEA metabolite formed by the action of 17β-hydroxy steroid dehydrogenase (17β-HSD), as a potent natural ligand activator of ERβ, whereas DHEA itself had no apparent activity when 17β-HSD was genetically knocked down. Possibly, in the experiments of Chen et al., DHEA was converted to 5-androsten -3β,17β-diol in their cell assays by 17β-HSD, a widely distributed enzyme, and this accounted for the ERβ transcriptional activity.

DHEA exerts broad-spectrum cancer chemopreventive activity in various mouse and rat models. In the two-stage TPA-promoted skin tumorigenesis model, as described previously, G6PD inhibition, leading to a reduction in inflammation and epidermal hyperplasia, is apparently the critical preventive mechanism. However, in other models, particularly prostate cancer chemoprevention [13], in which ERβ agonism has been shown to be prominent [68] and there is no strong inflammatory component [72], ERβ agonism by DHEA may play a significant preventive role.

## Identity of Sulfated Form of DHEA

Human clinical trials have provided no clear evidence of a beneficial effect of DHEA in treatment periods of up to two years using oral doses of 50 mg-75 mg [22]. The effective oral doses of DHEA used in the treatment of mice, rats, and rabbits, which produce cancer and atherosclerosis prevention, anti-obesity, anti-diabetic, and anti-GC effects, when extrapolated to the human, would indicate a human dose of 15X-30X the 75 mg dose [73]. The animal equivalent of the 75 mg human dose would produce no efficacy in preclinical studies. The rationale for using these DHEA doses in clinical trials is that they raise DHEA(S) levels in the elderly to the maximum levels found in the second decade [22].

DHEA exists in human serum almost exclusively as the sulfate, with a concentration of the sulfate ~400 X that of free DHEA [5]. When compared to DHEA, DHEA(S) is virtually inactive as a G6PD inhibitor [33], produces no apparent anti-GC effect in cultured human adipocytes [74], nor demonstrates any significant binding to ERβ [58]. It is generally assumed that DHEA(S) is a reservoir for DHEA, but there is little evidence to support this [75].

Over a period of about 15 years, Oertel and co-workers published a series of papers in which they described the isolation of DHEA sulfatide, a molecule in which DHEA sulfate is esterified to a diacyglycerol moiety, from human plasma [76]. They reported that DHEA sulfatide was the predominant sulfated form of DHEA in human plasma, and, in contrast to DHEA sulfate, which is virtually inactive as a G6PD inhibitor, DHEA sulfatide was a more potent G6PD inhibitor than DHEA [77]. Others have not corroborated Oertel’s findings, and the work has received little attention. Lieberman and co-workers, however, isolated DHEA-sulfolipid conjugates from rat, rabbit, and dog brain tissues, which were cleaved with triethylamine treatment, consistent with the properties of a steroid sulfatide [78]. The investigators concluded: “Failure to confirm Oertel’s results may be ascribed to the extreme lability of the conjugates, which may possibly be easily dissociated by treatment with even weak nucleophiles, such as water, alcohols, or heat, as is commonly used in the processing of tissues for analysis” [78].

We previously prepared synthetic DHEA sulfatide and confirmed that it is a more potent G6PD inhibitor than DHEA. We also found that the sulfatide, when injected ip in mice, was more potent than DHEA in suppressing TPA-stimulated epidermal DNA synthesis, whereas DHEA sulfate was inactive [79].

Very possibly the sulfated form of DHEA found in human plasma following oral administration of 50 mg-100 mg is DHEA sulfate, produced during first-passage liver metabolism, and is not identical to the predominant sulfated form which exists naturally in human plasma. Thus, these oral doses of DHEA may not be restoring DHEA(S) levels in the elderly to levels found in the second decade. Clearly, additional work is warranted to clarify if a biologically active sulfated form of DHEA predominates in human plasma.

## Non-Androgenic DHEA Analog

The clinical development of DHEA, using doses that are the human equivalent of therapeutically effective doses in preclinical studies, is compromised by DHEA’s androgenicity [18]. Androgens have numerous untoward effects. In women short term administration of the orally active androgen, methyltestosterone, worsened insulin sensitivity, an effect also seen with high dose (1600 mg) DHEA administered to post menopausal females for four weeks [80, 81]. Also, androgenicity, as determined by sex hormone binding globulin and free testosterone levels, is positively associated with cardiovascular risk factors in women [82, 83].

The androgenicity of DHEA, with a greater potential for adverse effects in females, may account for the differential association between high plasma DHEA(S) level and reduced mortality that has been observed in men but not in women [84]. In one of the largest, and presumably longest (27 years), prospective epidemiological studies in assessing plasma DHEA(S) levels with mortality, Enomoto et al. found that high DHEA(S) level was a highly significant predictor of longevity in men [2]. In contrast, in women there was no significant relationship, and there was also a possible signal of decreasing longevity with increasing DHEA(S) level (P=0.07).

In the male insulin-resistant, diabetic mouse (C57BLKs/J-db/db), which have very low testosterone levels, comparable to the female, there is a narrow window of anti-hyperglycemic efficacy following DHEA administration. DHEA, when administered at a dose of 200 mg/kg, significantly reduced fasting plasma glucose, yet when the dose was raised to 300 mg/kg, there was no reduction in glucose level, very probably as a result of the androgenic state induced at the higher dose [18].

DHEA treatment also raised plasma testosterone levels ~25 fold and produced a dose-related increase in seminal vesicle weights. In contrast, treatment with the non-androgenic analog, fluasterone, produced a normal dose-response, with a marked lowering of glucose levels at the highest dose, with no increase in seminal vesicle weights [18].

Fluasterone, when compared to DHEA, is also a more potent G6PD inhibitor [42] as well as a more potent anti-glucocorticoid [54], anti-diabetic [18] and anti-obesity [10,42] agent, and has at least comparable activity in cancer chemoprevention [10-13]. Fluasterone, like DHEA, is also about 40X as bioavailable when administered non-orally (subcutaneous, buccal, transdermal) as orally because of first-passage entero-hepatic metabolism (54 and Schwartz, A, Pashko, L, unpublished observation). Fluasterone is currently in clinical trials, and the development of such non-androgenic DHEA analogs may further our understanding of the potential therapeutic utility of this class of compounds.
